# Supplementary material for: Knowledge, attitude, and practice regarding dengue among non-health undergraduate students of Nepal
Source: PLoS Negl Trop Dis. 2024 May 28;18(5):e0012222. doi: 10.1371/journal.pntd.0012222 (PMC11161042; doi:10.1371/journal.pntd.0012222)
Supplement: S1 Questionnaire — (DOCX) [file pntd.0012222.s001.docx]

***Knowledge, Attitude and Practices regarding Dengue among non-health undergraduate students of Nepal***

*Namaste !!*

*You are most welcome to the survey. My name is Sheetal Bhandari. I am a student of Bachelors in Public Health final year at Central Department of Public Health, Institute of Medicine (IOM), Maharajgunj ,Kathmandu.We will carry out this research as follows. If you understand the purpose and content of the research and are willing to participate ,please proceed.*

*This survey is part of academic research designed to assess knowledge ,attitude and practice regarding dengue virus infection among non-health undergraduate students of Nepal. . It will take around 5 -10 minutes for you to complete this survey.*

*While we would certainly appreciate your understanding and participation in the survey, you can decide whether or not to participate in the survey. You will not suffer any penalty or disadvantage if you refuse to participate or decide to participate and later withdraw. Your participation is important for this study. Your information will help to plan various health education and promotion activities.*

*Confidentiality:*

*The information you provided will be very important for concerned authorities. We assure you that the information you provided will be kept completely confidential. We promise to protect your confidentiality.*

*Ethical approval:*

*Ethical Approval of the study has been taken from Institutional Review Committee-IOM. A letter of support has been received from the Central Department of Public Health, IOM.*

*Potential risks and Discomforts*

*There are no foreseeable risks in participating in this survey.*

*For any queries, you can contact me at +977 9867710846*

*[sheetal.bhandari07@gmail.com](mailto:sheetal.bhandari07@gmail.com)*

| ***Do you voluntarily consent to take part in this study?***   - ***Yes*** - ***No*** |
| --- |

**KAP Questionnaire**

1. **Demographic characteristics of respondents**

Age:

Sex: Male … Female …. Others ….

Marital Status: Single …., Married …. Divorced …., Widow

Education:

Illiterate … Read/write ….. Primary …. Secondary …. Intermediate ….. Graduate ….. Postgraduate ……

Annual Income: ….

1. **Knowledge of Dengue vectors**

| **S.N.** | **Variables** | **Yes** | **No** | **Do not know** |
| --- | --- | --- | --- | --- |
| **1** | Have you heard about dengue?  ( Continue the interview if s/he has heard about it) |  |  |  |
|  | **Knowledge of symptoms** |  |  |  |
| **2** | Is fever a symptom of dengue? |  |  |  |
| **3** | Is headache a symptom of dengue fever? |  |  |  |
| **4** | Is joint pain a symptom of dengue fever? |  |  |  |
| **5** | Is muscle pain a symptom of dengue fever? |  |  |  |
| **6** | Is pain behind the eyes a symptom of dengue fever? |  |  |  |
| **7** | Are nausea/vomiting symptoms of dengue fever? |  |  |  |
| **8** | Is rash a symptom of dengue fever? |  |  |  |
| **9** | Is headache a symptom of dengue fever? |  |  |  |
| **10** | Is diarrhea common in dengue fever? |  |  |  |
| **11** | Is back pain common in dengue fever? |  |  |  |
| **12** | Is stomach pain common in dengue fever? |  |  |  |
|  |  |  |  |  |
|  | **Knowledge of transmission** |  |  |  |
| **13** | Can all mosquitoes transmit dengue fever? |  |  |  |
| **14** | Do the *Aedes* mosquitoes transmit dengue fever? |  |  |  |
| **15** | Do flies transmit Dengue fever? |  |  |  |
| **16** | Do ticks transmit Dengue fever? |  |  |  |
| **17** | Does ordinary person to person contact transmit Dengue fever? |  |  |  |
| **18** | Is Dengue fever transmitted through food and water? |  |  |  |
| **19** | Can dengue fever be transmitted by blood transfusion? |  |  |  |
| **20** | When are the Dengue mosquitoes likely to feed/bite? |  |  |  |
|  | Night time |  |  |  |
|  | Day time |  |  |  |
|  | Both day and night |  |  |  |
| **21** | Mosquitoes breed in standing water |  |  |  |
|  | Yes |  |  |  |
|  | No |  |  |  |
|  | Do not know |  |  |  |
| **22** | Window screens and bed net reduce mosquitoes |  |  |  |
|  | Yes |  |  |  |
|  | No |  |  |  |
|  | Do not Know |  |  |  |
| **23** | Insecticide sprays reduce mosquitoes and prevent Dengue |  |  |  |
| **24** | Tightly covering water containers reduces mosquitoes |  |  |  |
| **25** | Removal of standing water can prevent mosquito breeding |  |  |  |
| **26** | Mosquito repellents prevent mosquitoes |  |  |  |
|  |  |  |  |  |
|  | **Knowledge of *Aedes* Mosquitoes** |  |  |  |
| **27** | Can you identify *Aedes* mosquitoes? |  |  |  |
|  |  |  |  |  |
| **28** | **Sources of Information on dengue** |  |  |  |
|  | Radio |  |  |  |
|  | Television |  |  |  |
|  | Health Professionals |  |  |  |
|  | Miking |  |  |  |
|  | Neighbors |  |  |  |
|  | Teachers |  |  |  |
|  | Children |  |  |  |
|  | Others |  |  |  |

1. **Attitudes towards dengue**
2. **Is Dengue fever a serious illness?**

*Strongly agree*

*Agree*

*Disagree*

*Strongly Disagree*

*Not sure*

1. **Are you at risk of getting dengue?**

*Strongly agree*

*Agree*

*Disagree*

*Strongly Disagree*

*Not sure*

1. **Can Dengue fever be prevented?**

*Strongly agree*

*Agree*

*Disagree*

*Strongly Disagree*

*Not sure*

1. **Is controlling the breeding places of mosquitoes a good strategy to prevent dengue fever?**

*Strongly agree*

*Agree*

*Disagree*

*Strongly Disagree*

*Not sure*

1. **Do you think that stagnant water around the houses in discarded tires, broken pots and bottles are breeding places of *Aedes* mosquitoes?**

*Strongly agree*

*Agree*

*Disagree*

*Strongly Disagree*

*Not sure*

1. **Do you think communities should actively participate in controlling the vectors of Dengue?**

*Strongly agree*

*Agree*

*Disagree*

*Strongly Disagree*

*Not sure*

1. **Which prevention against dengue fever do you practice?**

| **S.N.** | **Variables** | **Yes** | **No** | **Do not know** |
| --- | --- | --- | --- | --- |
| 1 | Prevent mosquito-man contact |  |  |  |
| 2 | Use insecticide sprays to reduce mosquitoes |  |  |  |
| 3 | Use professional pest control to reduce mosquitoes |  |  |  |
| 4 | Use screen windows to reduce mosquitoes |  |  |  |
| 5 | Eliminate standing water around the house to reduce mosquitoes |  |  |  |
| 6 | Cut down bushes in the yard to reduce mosquitoes |  |  |  |
| 7 | Prevent water stagnation |  |  |  |
| 8 | Use mosquito eating fish to reduce mosquitoes |  |  |  |
| 9 | Use mosquito coils to reduce mosquitoes |  |  |  |
| 10 | Cleaning of garbage/ trash |  |  |  |
| 11 | Disposing water holding containers such as tires, parts of automobiles, plastic bottles, crack pots etc. |  |  |  |
| 12 | Use Mosquito Repellent/ cream |  |  |  |
| 13 | Use of fan |  |  |  |
| 14 | Use of smoke to drive away mosquitoes |  |  |  |
| 15 | Covering body with clothes |  |  |  |
| 16 | Do nothing to reduce mosquitoes |  |  |  |
| 17 | Eliminating mosquito breeding sites |  |  |  |
| 18 | Cover water containers in the home |  |  |  |
| 19 | Frequently cleaning water filled containers and ditches around the house |  |  |  |
|  | *Always* |  |  |  |
|  | *Often* |  |  |  |
|  | *Sometimes* |  |  |  |
|  | *Never* |  |  |  |
| 20 | Government sprays insecticides for controlling mosquitoes |  |  |  |
| 21 | Turning containers upside down to avoid water collection |  |  |  |
