# Supplementary material for: Knowledge, attitude, and practice regarding dengue among non-health undergraduate students of Nepal
Source: PLoS Negl Trop Dis. 2024 May 28;18(5):e0012222. doi: 10.1371/journal.pntd.0012222 (PMC11161042; doi:10.1371/journal.pntd.0012222)
Supplement: Attachment — Submitted filename: Reviewer Response.docx [file pntd.0012222.s002.docx]

**Title: Knowledge, attitude and practice regarding dengue among non-health undergraduate students of Nepal.**

The Editorial Board,

PLOS-Neglected Tropical Diseases

**Thank you for the review and for providing feedback/suggestions.**

We have addressed the comments and made necessary changes/corrections in the updated manuscript entitled **“Knowledge, attitude and practice regarding dengue among non-health undergraduate students of Nepal.” [Ms. Ref. No: PNTD-D-23-01001].** Both tracking and a clean version of an updated version of the manuscript are also provided along with this response.

Thank you.

Sincerely,

Manish Rajbanshi

Corresponding author

Methods

Reviewer #1

**Sample size calculation: How did the authors confirm equal participation from the provinces or consider other factors for the selection of samples to generalize the results?**

Since the study was conducted using convenience sampling. So, the number of participants was not based on the equal participation from the provinces in this study. Further, there may only be very little difference in the KAP of students differentiated by Provinces in Nepal.

**Discuss briefly about the sample size calculation using the formula with a non-response rate.**

The sample size was calculated using the formula N=Z^2^pq/d^2^ (Cochran’s Formula). Assuming 50% prevalence (p) from a previous study, 5% margin of error (d), 95% confidence interval (CI), and 10% non-response rate.

The sample size (n) is calculated according to the formula: N=Z^2^pq/d^2^

Where: Z = 1.96 for a confidence level (α) of 95%, p = proportion (expressed as a decimal), d = margin of error.

Z = 1.96, p = 0.5, d = 0.05

n = 1.962 * 0.5 * (1 - 0.5) / 0.052

n = 0.9604 / 0.0025 = 384.16

n ≈ 385

The sample size is equal to 385.

Adding 10% non-response rate the actual number of participants becomes 385 + 10% of 385 = 423.5 (i.e., 424 participants).

**Study population age group: is it 18-22 or up to 25?**

The study population age group is not 18-22 or 18-25. The study population was non-health undergraduates who were above 18 years of age.

**Ethical clearance: is the clearance from the Institute of Medicine, Tribhuvan University is applicable for all university? What about NHRC clearance for this study?**

The Institution Review Committee of Institute of Medicine, Tribhuvan University is a listed Review Committee of the Nepal Health Research Council (NHRC).

The NHRC Ethical Review Board (ERB) cannot possibly review and monitor all research being conducted in the country. Thus, the ERB has delegated its power to Institutional Review Committees (IRCs) established at healthcare facilities, academic institutions and research institutions throughout Nepal.

NHRC recommends for all research (except for multi-country, externally funded, clinical trials) instead of seeking approval from NHRC, ethical approval for studies be obtained from the local Institutional Review Committee (IRC) of the researcher in institutions where the IRC is already established.

**Results**

**Reviewer #1**

**I suggest to highlights the important result findings of table rather than explaining too much below the particular table.**

Thank you for the suggestion. We’ve tried to implement the suggestion in our updated tracked-changed manuscript.

**P< 0.05 significant value indicates by asterisk rather than bold in table.**

Thank you for the suggestion. We’ve tried to implement the suggestion in our updated tracked-changed manuscript.

**Please construct the 2×2 table throughout the manuscript as far as possible including table 5**

Thank you for the suggestion. We’ve tried to implement the suggestion in our updated tracked-changed manuscript.

**There are too many irrelevant tables. It is suggested to remove tables if there are no significant results including table 5**

Thank you for the suggestion. We’ve tried to implement the suggestion in our updated tracked-changed manuscript.

**Fig 1: It is suggested for further formatting**

Thank you for the suggestion. We’ve tried to implement the suggestion in our updated tracked-changed manuscript.

**It is somehow difficult to understand the knowledge in relation to marital status? Could you explain?**

Thank you for the opportunity to make our manuscript better. We added “This study demonstrated that married groups had a better level of knowledge than their counterparts. It could be that married couples often share their daily experiences including health information, and if one partner has access to health-related information or works in the health sector, they may share their knowledge with family members” in the revised manuscript.

**Discussion need rewriting to discuss finding in relation to previous publication and highlighting the major issues to be focused since the disease is first reported in 2004.**

Thank you for the suggestion. We’ve tried to implement the suggestion in our updated tracked-changed manuscript.

**It is also suggested to have clear recommendation for the local and national government based on the finding**

Thank you for the suggestion. We’ve now incorporated recommendations for the local and national government based on the finding.

**Summary and general comments**

**Reviewer #1**

**Major comments: This MS was prepared based on a web-based interview with a non-health undergraduate student from four universities in Nepal. I wonder why the four universities selected the undergraduate non health group for this study. The other point is the language of the questionnaire: is it English or Nepali? I could find if the author performed pretesting and validation of the questionnaire. The questions are so specific that they can only be understood by health workers, and these seem to be leading questions that have different outcomes. Have you defined non-health group and health groups? Justification for adding value to this paper in relation to previous publications, including Meghnath et al. and others? There are lots of redundancies and duplication in the MS and the tables. The other important and pertinent issue is that there is not much emphasis on climatic factors including temperature, precipitation, or rainfall.**

Undergraduate non-health groups were the group of concern for this study as health group students are more likely to receive dengue-related information than their counterparts. So this study was conducted to explore the situation among non-health undergraduates.

The questionnaire of the study was in English language.

Pretesting of the questionnaire was done among around 10% (45 participants) of the total sample size to check the feasibility of the study tool. The Cronbach alpha coefficient was obtained 0.80, 0.72, and 0.81 for the knowledge, attitude and practice respectively which was similar to the study conducted by Dhimal et al. in 2014.

During pretesting no major problems in the understanding of the questionnaire (or any technicalities) were reported from the study participants. Moreover, the tool was adapted from the previous study conducted in Nepal (Dhimal et al. 2014).

This study included participants from engineering, agriculture, management, forestry, veterinary, arts, and information technology educational backgrounds (non-health background). The students belonging to the medical, public health, nursing or para-medical subjects (like pharmacology, medical lab technology, and radiology) were counted as students from health backgrounds.

Similar studies were conducted among general communities in Nepal but the study has not been carried out among undergraduate non-health students. This study particularly tried to capture the situation of the knowledge, attitude and practice of non-health undergraduate students. The study findings will help to identify the gaps and what interventions could be planned to better their understanding and practice in an endemic condition of dengue.

Thank you for the comments regarding the redundancies in the manuscript. We have tried to address the problem of redundancies in our updated track-changed manuscript.

This study had limited emphasis on climatic factors including temperature, precipitation, rainfall which will be mentioned in the limitation of the study in the updated track-changes manuscript.

**Minor comments**

**Write the complete form of KAP in the initially in the abstract.**

Thank you for sharing. We have addressed the comments and made the necessary changes in the updated manuscript.

**Page 4, line 68 mentioned focused awareness including street families in Nepal. I am not aware of the such families in Nepal! can author clarify.**

Thank you for your comments, we agree that street families in Nepal are not a reality. We have accordingly removed the line in the manuscript.

**Typographical error on page line 121 term strongly used twice? Needs appropriate correction.**

Thank you for your feedback. Changes have been incorporated accordingly.

**Line 149-150 error 94.2% married does not correlate with the Table 1.**

Thank you for your suggestion. Changes have been incorporated accordingly.

**The meaning is not understandable on page 20 line 216. It is suggested to rewrite.**

Thank you for your suggestion. Changes have been made accordingly.

**Grammatical errors should be addressed properly.**

Thank you for the suggestion. We’ve tried to implement the suggestion in our updated tracked-changed manuscript carefully.

**Reviewer#2**

**The study is important as Dengue is considered endemic in Nepal and is associated with significant socio-economic burden for the country. The study is well conducted but few issues need to be considered before publication.**

**As the sampling was convenience, how many students were approached for the study? What was the response rate?**

Thank you for providing an opportunity for sharing.550 participants were approached to fulfill the required number of sample size in this study. So, the response rate for this study was 78%.

**The analysis consists of several univariate analyses using chi-square test and correlation. Multivariate analysis using logistic regression would be more appropriate to identify independent predictors of knowledge, attitude and practice.**

Thank you for the suggestion. This study performed multivariate analysis for the prediction between independent variables and KAP. However, there was no association between the variables so we preferred to remove the results of multivariate analysis.

**In the discussion section, it would be good to discuss the stigma associated with disease regarding common misconception that the disease can be spread from person to person.**

Thank you for the suggestion. We’ve tried to implement the suggestion in our updated tracked-changed manuscript.

**The limitation of convenience sampling should be discussed in the limitation section.**

Thank you for the suggestion. We’ve tried to implement the suggestion in our updated tracked-changed manuscript.

**The questionnaire needed to be provided in the supplementary file.**

Thank you for the suggestion. The questionnaire will be provided as a supplementary file.
