# Supplementary material for: Knowledge, attitude, and practice regarding dengue among non-health undergraduate students of Nepal
Source: PLoS Negl Trop Dis. 2024 May 28;18(5):e0012222. doi: 10.1371/journal.pntd.0012222 (PMC11161042; doi:10.1371/journal.pntd.0012222)
Supplement: Attachment — Submitted filename: Review to PlOS NTD.docx [file pntd.0012222.s003.docx]

Thank you for addressing most of my suggestions; the authors have incorporated these changes. However, I still have some additional suggestions for the following areas to further enhance the manuscript.

1. In line 67, the 2023-12-15 dengue situation reported by EDCD on Dengue is updated as follows: Dengue Situation: Reported Dengue cases: 51243 Confirmed Deaths: 20 Affected Districts: 77 Number of Districts with > 500 cases: 18. <https://edcd.gov.np/news/20231215-dengue-situation-update>
2. In line 68, the ongoing response of the Government of Nepal (GON) towards vector control, including dengue, needs improvement. Please elaborate.
3. I am curious about how the author assumes that the level of Knowledge, Attitudes, and Practices (KAP) is not significantly different among the provinces, given the substantial variation in education levels between them.
4. The Ethics Review Committee (ERC) of the Institute of Medicine, Tribhuvan University, is authorized to grant ethics clearance for research proposals submitted by researchers affiliated with the institution and associated with a specific Institutional Review Committee (IRC). However, I am still uncertain whether this clearance extends to researchers from other universities (such as Kathmandu University, Pokhara University, and Purbanchal University) that are not part of IOM, TU.
5. Due to the absence of questionnaires addressing climatic and environmental factors as diseases progressed from their initial report in Chitwan, Nepal, in 2004, and now spanning two decades with cases reported in almost all 77 districts of Nepal, I suggest that the authors discuss dengue and its correlation with climate. Additionally, they could explore the low numbers of cases during the COVID-19 pandemic period, citing relevant references.
