# Supplementary material for: Knowledge, attitude, and practice regarding dengue among non-health undergraduate students of Nepal
Source: PLoS Negl Trop Dis. 2024 May 28;18(5):e0012222. doi: 10.1371/journal.pntd.0012222 (PMC11161042; doi:10.1371/journal.pntd.0012222)
Supplement: Attachment — Submitted filename: Tracked version_KAP_Nepal.docx [file pntd.0012222.s004.docx]

**Knowledge, attitude, and practice regarding dengue among non-health undergraduate students of Nepal**

Sheetal Bhandari^1^, Manish Rajbanshi^1^, Nabin Adhikari^1^, Richa Aryal^2^, Kshitij Kunwar^1^, Rajan Paudel^1,3^

**Affiliations**

^1^Central Department of Public Health, Institute of Medicine, Tribhuvan University, Kathmandu, Nepal

^2^Department of Public Health, Om Health Campus, Purbanchal University, Kathmandu, Nepal

^3^Unit of Health Sciences, Faculty of Social Sciences, Tampere University, Finland

**Corresponding author**

**Manish Rajbanshi**

Email: manishrajbanshi717@gmail.com

**Abstract**

Dengue poses a significant public health concern worldwide. It is identified a recentt emerging infectious diseases in Nepal. Understanding the dynamic between knowledge, attitudes, and practices related to dengue among students is crucial for effective prevention and control strategies. This study aimed to assess the Knowledge, Attitude and Practice (KAP) and associated factors of dengue virus infection among non-health undergraduate students.

A web-based cross-sectional study was conducted among 429 non-health undergraduate students at four prominent Nepalese Universities. Self-administered Googleonline forms were sent online for data collection. Data was cleaned and then exported to Statistical Package for the Social Sciences (SPSS) version20 (IBM) for analysis. Respondents' socio-economic and demographic characteristics were described using frequencies, percentages, median, and interquartile ranges. Chi-square tests examined the association between KAP and respondent’s characteristics, while Pearson's correlation coefficient determined the association between knowledge-attitude, attitude-practice, and knowledge-practice. Significance was determined at p < 0.05.

A total of 429 students participated in this study. Among them, half of the participants were females (50.3%). We found that 84.8% of participants had good knowledge while only 25.9% and 31.7% of participants had good attitude and practice regarding dengue respectively. Age (OR=1.9, CI: 1.1-3.4), marital status (OR=0.2, CI:0.1-0.6) and educational level (OR=3.4, CI:1.8-6.5) were significantly associated with knowledge regarding dengue. Attitude was not associated with any demographic or socioeconomic characteristics of respondents. Only age (OR=1.6, CI: 1.0-2.4) was significantly associated with practice. The knowledge-attitude (r_ka_ = 0.01, P< 0.05), and knowledge-practice (r_kp_ = 0.20, P < 0.05) were positively correlated while attitude-practice (r_ap_ = -0.12, P< 0.05) were negatively correlated in this study.

This study highlights the need to improve the knowledge, attitude, and practice (KAP) of undergraduate non-health students regarding dengue prevention and control and shows that students with good knowledge on dengue do not necessarily have good preventive practices also. It suggests various ways to achieve this including school health education, social media, targeted interventions, and community involvement. National government has to guide and support local governments and other stakeholders to promote further studies on the factors affecting dengue transmission and prevention in light of the current demographic transitioning of dengue.

***Keywords: dengue, knowledge, attitude, practice, Nepal***

**Introduction**

Dengue is considered one of the predominant arboviral infections caused by serotypes (DENV 1-4) and transmitted through bites of infected *Aedes aegypti* (1). In the last 50 years, the incidence of dengue has increased 30 times with increasing geographic expansion and has become a major public health concern globally (2). Around 70% of dengue cases were reported in the Asian Region (3). Dengue is endemic in more than 100 countries, mostly among the tropical and subtropical populated areas. It is being reported predominantly in the South-East Asia Region (SEAR), the Americas and the Western Pacific region (4).

Dengue has been identified as a recent emerging infectious diseases in Nepal. A substantial increase in dengue incidence has been challenging to the health system of Nepal for the prevention and control of dengue (5). Nepal had its first dengue outbreak in 2006 followed by the major outbreak in 2010, 2013, and 2016 (6). Tracking cyclic outbreaks, 18,000 cases were reported in 68 districts in 2019 (7). In 2022, there had been 54,784 reported dengue cases and 88 deaths which were almost three times higher compared to 2019 marking the largest number ever recorded in the country. As of 15 July, 2023, 2,930 dengue cases had been identified from 68 districts and 8 deaths (8). The ongoing response of the Government of Nepal (GoN) towards dengue including vector control needs to be improved (9).

Previous studies among non-health students showed that their knowledge of dengue was poor (11). University students represent an engaged demographic group to gauge knowledge, perceptions, and practices against vector-borne diseases like dengue. Additionally, university students can be a good source for community preparedness that will help to share knowledge with families and establish sound dengue fever preventive and control practices for the community as a whole. We view undergraduate non-health students as a potential target population to tackle the growing dengue crisis, environmental control and community engagement including students are the best measures for prevention and control. Previous studies have focused community member or health related students, the latter likely to have more knowledge than the general public.

This study particularly tried to capture the situation of the knowledge, attitude and practice of non-health undergraduate students and to possibly help identify the gaps and what interventions could be planned to better their understanding and practice in an endemic condition of dengue. The findings of the study may be the point of reference to bridge the gap to design interventional strategies in collaboration with educational institutions, community, local health authorities, and policymakers to establish effective vector control programs and to prepare for dengue outbreaks in the future.

**Methods**

**Study design and setting:** A web-based cross-sectional study was employed across all the provinces of Nepal. Data collection was carried out between January to April of 2020.

## Study population

The study population was non-health undergraduates who were above 18 years. This study included participants from engineering, agriculture, management, forestry, veterinary, arts, and information technology backgrounds. It included participants from the top four universities in Nepal: Tribhuvan University, Kathmandu University, Pokhara University, and Purbanchal University of Nepal.

## Sample size and sampling technique

Convenience sampling was adopted to recruit the participants for data collection. Sample size was calculated using the formula N=Z^2^pq/d^2^ (12). Assuming the 50% prevalence (p) from the previous study (13), 5% margin of error (d), 95% confidence interval (CI), and 10% non-response rate, the total number of participants for this study was determined as 424 in this study.

**Data collection**

A self-administered online Google form in English was sent via online for data collection. One participant could only fill out the formonce in this study.

**Measures**

The study tool was adapted from a similar study conducted by Dhimal et.al in Nepal (14). The KAP questionnaire consists of two sections, the first section is related to the socio-economic and demographic characteristics of participants which includes: age (in years), sex (male/female), marital status (married/unmarried), ethnicity (Brahmin/Chhetri, Janajati, Madhesi, Dalit, Muslims, and others), education level (first year, second year, third year, and fourth year), family monthly income (<NRs.40,000 and ≥NRs.40,000) and Province(Koshi Province, Madhesh Province, Bagmati Province, Gandaki Province, Lumbini Province, Karnali Province and Sudurpaschim Province).

The second section consisted of questions related to the Knowledge, Attitude and Practice (KAP) of dengue (supplementary text S1). Altogether there were a total of 48 questions in this section. It includes 23 questions on knowledge regarding signs/symptoms, mode of transmission and preventive practices on dengue.

In the attitude section, 6 questions were on serious illness, risk and prevention of dengue, strategies to prevent dengue, the breeding place of mosquitoes, and active participation of the community in the prevention of dengue. A 5-point Likert scale (strongly agree, agree, disagree, strongly disagree and not sure) was used to assess the attitude. While 19 questions were asked on the preventive practice towards dengue among participants.

Evaluation of knowledge, attitude and practices was done by assigning ‘1’ for correct /positive response and ‘0’ for incorrect/negative response. Participants were categorized into having good knowledge, good attitude and good practice if they had scored 80% or above, and poor if they had scored below 80% in this study (14).

**Data management and analysis:** Data collected online was downloaded as a spreadsheet. All the collected information was systematically compiled, coded, checked and edited before exporting to Statistical Package for the Social Sciences (SPSS) version 20 (IBM) for analysis. The respondents’ socio-economic and demographic characteristics were described using frequencies, percentages, median and interquartile ranges. Chi-square tests were performed to establish the association of KAP and individual characteristics as appropriate. Pearson coefficient of correlation was used to determine the relationship between knowledge-attitude, attitude-practice, and knowledge-practice. The statistical significance was set at p-value <0.05.

**Ethical approval:** The approval was obtained from the Institutional Review Committee of the Institute of Medicine (IOM), Tribhuvan University, Nepal (Reference no; 216(6-11) E2 077/078). Informed consent was obtained from participants on the first page of the online questionnaire. Study objectives were clearly explained on the online form to the study participants before data collection.

**Results**

**Individual characteristics of the participants**

Of 550 participants receiving a request to participate in our KAP survey, 429 responded (78% response rate). An almost equal proportion of females (50.3%) and males (49.7%) participated in this study. More than half of the participants (54.1%) belonged to the age group 22 or above. The majority of participants (71.8%) belonged to Brahmin/Chhetri ethnic group followed by Janajati (15.6%). Nearly all of the participants (94.2%) were unmarried . The fourth year bachelor students were represented the highest proportion participants (38.2%), followed by first year (28.9%), third year (19.6%) and second year (13.3%).

In this study, the majority of the participants (63.2%) belonged to the families having a monthly income of below 303.03 USD. Most participants were from Lumbini Province (41.5%) followed by Bagmati Province (24.7%). (Table 1)

*Table 1: Individual characteristics of the participants*

| **Characteristics** | **Numbers (n)** | **Percentage (%)** |
| --- | --- | --- |
| **Sex** |  |  |
| Female | 216 | 50.3 |
| Male | 213 | 49.7 |
| **Age group (in years)**  **Median ± IQR= (22.0±3.0)** |  |  |
| Less than 22 | 197 | 45.9 |
| 22 or above | 232 | 54.1 |
| **Ethnicity** |  |  |
| Brahmin/Chhetri (31.25%, according to Wikapedia) | 308 | 71.8 |
| Janajati (30.81%) | 67 | 15.6 |
| Madhesi (23.43%) | 24 | 5.6 |
| Dalit (8.12%) | 12 | 2.8 |
| Others (Marwari,Bangali) (0.32%) | 18 | 4.2 |
| **Marital status** |  |  |
| Unmarried | 404 | 94.2 |
| Married | 25 | 5.8 |
| **Educational level** |  |  |
| First year | 124 | 28.9 |
| Second year | 57 | 13.3 |
| Third year | 84 | 19.6 |
| Fourth year | 164 | 38.2 |
| **Monthly income of the family (in NRs)** |  |  |
| Below or equal to NRs. 40000 (≤USD 303.03) | 267 | 63.2 |
| Above NRs. 40000 (>USD 303.03) | 162 | 37.8 |
| **Province** |  |  |
| Koshi Province | 61 | 14.2 |
| Madhesh Province | 33 | 7.7 |
| Bagmati Province | 106 | 24.7 |
| Gandaki Province | 20 | 4.7 |
| Lumbini Province | 178 | 41.5 |
| Karnali Province | 18 | 4.2 |
| Sudurpaschim Province | 13 | 3.0 |

**Source of information on dengue**

The majority of the participants (16.2%) had heard of dengue from television followed by social media (15.6%). (Fig 1)

*Figure 1: Source of information on dengue*

**Knowledge on dengue among the participants**

The majority of the participants recognized fever (90%), headache (67.1%) and rash (59.7%) were the major signs/symptoms of dengue. While only 13.5 % incorrectly stated all mosquitoes can transmit dengue. Around (67%) were aware that *Aedes* mosquitoes transmit dengue. This study found 43.6% and 34.5% of the participants believed dengue can be transmitted by flies/ticks and from the infected person with dengue, respectively. Participants correctly observed that dengue can be transmitted by blood transfusion (71.3%) and but 46.6% incorrectly though transmission occurred through contaminated water and food. The majority of the participants were aware of the way of reducing mosquitoes by using mosquito screen and bed net (80.7%) and using insecticide sprays (81.4%) (Table 2).

*Table 2: Knowledge on dengue among the participants. Incorrect responses indicated in red.*

| **Statements** | **Correct response, n (%)** |
| --- | --- |
| **Knowledge on signs/symptoms** |  |
| Fever | 386 (90.0) |
| Headache | 288 (67.1) |
| Rash | 256 (59.7) |
| Nausea/vomiting | 219 (51.0) |
| Joint pain | 213 (49.7) |
| Muscle pain | 190 (44.3) |
| Pain behind eyes | 172 (40.1) |
| Back pain | 90 (21.0) |
| Diarrhea | 86 (20.0) |
| Stomach pain | 61 (14.2) |
| **Knowledge on Transmission** |  |
| All mosquito transmits dengue fever | 58 (13.5) |
| Aedes mosquito transmit dengue fever | 286 (66.7) |
| Flies and ticks transmit dengue fever | 187 (43.6) |
| Does ordinary person to person contact transmit Dengue fever | 148 (34.5) |
| Transmitted through food and water | 200 (46.6) |
| Transmitted by blood transfusion | 306 (71.3) |
| Mosquito breed in standing water | 365 (85.1) |
| Mosquito screen and bed net reduce mosquito | 346 (80.7) |
| Insecticide sprays reduce mosquito and prevent dengue | 349 (81.4) |
| Tightly covering water containers reduce mosquitoes | 334 (77.9) |
| Removal of standing water can prevent mosquito breeding | 378 (88.1) |
| Mosquito repellants prevent mosquito bite | 313 (73.0) |
| Identify *Aedes* mosquito | 167 (38.9) |
| Dengue mosquito feeding time |  |
| Day time | 192 (44.8) |
| Night time | 237 (55.2) |

**Factors associated with the knowledge on dengue among the participants**

Table 3 demonstrated 15.2 % of the participants had a poor knowledge on dengue. This study showed knowledge was significantly associated with age (OR=1.9, CI:1.1-3.4), marital status (OR=0.2, CI :0.1-0.6), and educational level (OR=3.4, CI:1.8-6.5).

***Table 3: Factors associated with the knowledge on dengue among the participants***

| **Characteristics** | **Knowledge** | | **P-value** | **Odds ratio**  **(95% CI)** |
| --- | --- | --- | --- | --- |
|  | **Good**  **n (%)** | **Poor**  **n (%)** |  |  |
| **Level of knowledge** | 364 (84.8) | 65 (15.2) |  |  |
| **Age** |  |  |  |  |
| Less than 22 | 176 (89.3) | 21 (10.7) | 0.01* | 1.9 (1.1-3.4) |
| 22 or above | 188 (81.0) | 44 (19.0) |  |  |
| **Sex** |  |  |  |  |
| Female | 185 (85.6) | 31 (14.4) | 0.6 | 1 (1.9-0.6) |
| Male | 179 (84.0) | 34 (16.0) |  |  |
| **Marital Status** |  |  |  |  |
| Unmarried | 16 (64.0) | 9 (36.0) | 0.03* | 0.2 (0.1-0.6) |
| Married | 348 (86.1) | 56 (13.9) |  |  |
| **Education level** |  |  |  |  |
| Not completed two years | 168 (92.8) | 13 (7.2) | <0.001** | 3.4 (1.8-6.5) |
| Completed at least two years | 196 (79.0) | 52 (21.0) |  |  |
| **Monthly income of the family** |  |  |  |  |
| Below or equal to NRs. 40000 (≤USD 303.03) | 123 (89.8) | 14 (20.8) | 0.06 | 1.8 (0.9-3.4) |
| Above NRs. 40000 (>USD 303.03) | 241 (82.5) | 51 (17.5) |  |  |

*P-value statistically significant at the level of 0.05

**Attitude on dengue among the participants**

In this study, more than half of the participants (55.5%) agreed that dengue is a serious disease. While one-third (32.4%) agreed that they were at risk of getting dengue. More than half of the participants (54.1%) believed that dengue fever can be prevented. Half of the participants (47.8%) strongly agreed that controlling the breeding place is a good strategy to prevent dengue. While 45% of the participants strongly agreed that dengue breeds in the stagnant water of broken pots and bottles (Table 4).

*Table 4: Attitude on dengue among the participants*

| ***Statements*** | **Strongly agree** | **Agree** | **Disagree** | **Strong disagree** | **Not sure** |
| --- | --- | --- | --- | --- | --- |
|  | **n (%)** | **n (%)** | **n (%)** | **n (%)** | **n (%)** |
| Dengue fever a serious disease | 160 (37.3) | 238 (55.5) | 17 (4.0) | 2 (0.5) | 12 (2.8) |
| Risk of getting dengue | 33 (7.7) | 139 (32.4) | 82 (19.0) | 25 (5.8) | 150 (35.0) |
| Dengue fever can be prevented | 165 (38.5) | 232 (54.1) | 5 (1.2) | 0 (0) | 27 (63.0) |
| Good strategy to prevent dengue is controlling the breeding place | 205 (47.8) | 186 (43.4) | 13 (3.0) | 4 (0.9) | 21 (4.9) |
| Stagnant water in broken pots/bottles are breeding places for Aedes | 193 (45.0) | 186 (43.4) | 9 (2.1) | 3 (0.7) | 38 (8.9) |
| Communities are responsible for controlling the vector of dengue | 269 (62.7) | 136 (31.7) | 6 (1.4) | 2 (0.5) | 16 (3.7) |

**Factors associated with attitude on dengue among the participants**

In this study, almost three-fourths of the participants (74.1%) had a poor attitude on dengue. The attitude was not found to be associated with age group (OR=1.2, CI: 0.7-1.8), sex (OR=1.1, CI:0.7-1.7), marital status (OR=1.1, CI:0.4-2.7), education (OR=0.4, CI:0.5-1.2), and monthly income of the family (OR=0.6, CI:0.4-1.1) .

**Practice on dengue among the participants**

Table 5 shows the frequency of use of common denge prevention practices including preventing human/mosquito contact (73.2%), use of insecticide spray (77.9%), use of window screens (84.4%), eliminating standing water around the house (85.8%), cutting down the bushes in the yard (83.9%), using mosquito coils/repellent cream (82.1%), garbage removal (92.1%), dispose of water containing containers (90.2%), and covering body with clothes (88.6%). Unfortunately, 14% believed doing nothing can reduce mosquitoes.

*Table 5: Practice on dengue among the participants*

| ***Statements*** | **Correct response n (%)** |
| --- | --- |
| Use insecticide spray | 334 (77.9) |
| Prevent mosquito man contact | 314 (73.2) |
| Use professional pest control | 294 (68.5) |
| Use window screens | 362 (84.4) |
| Eliminate standing water around the house | 368 (85.8) |
| Cut down bushes in the yard | 360 (83.9) |
| Using mosquito eating fish | 216 (50.3) |
| Using mosquito's coils/mosquito repellent cream | 352 (82.1) |
| Removal of garbage/trash | 395 (92.1) |
| Disposing water holding containers such as tires, parts of automobiles, plastic bottles, crack pots etc. | 387 (90.2) |
| Use of fan to drive repelling mosquitoes | 269 (62.7) |
| Use of smoke to drive away mosquitoes | 206 (48.0) |
| Covering body with clothes | 380 (88.6) |
| Do nothing to reduce mosquitoes | 60 (14.0) |
| Eliminating mosquito breeding sites | 373 (86.9) |
| Covering water containers in house | 391 (91.1) |
| Government spray insecticide for controlling mosquitoes | 270 (62.9) |
| Turning containers upside down to avoid water collection | 309 (72.0) |
| Frequently cleaning water filled containers and ditches around the house |  |
| Always | 44 (10.3) |
| Often | 86 (20.0) |
| Sometimes | 290 (67.6) |
| Never | 9 (2.1) |

**Factors associated with practice on dengue among the participants**

Table 6 shows more than two-thirds of the participants (68.3%) had good practice in this study. Only age (OR=1.6, CI:1.0-2.4) was significantly associated with the practice. The other characteristics sex (OR=1.6, CI:0.7-1.7), marital status (OR=1.4, CI:0.6-3.3), educational level (OR=1.3, CI:0.9-2.1) and monthly income of the family (OR=0.7, CI:0.4-1.1) were not associated with the practice in this study.

***Table 7: Factors associated with practice on dengue among the participants***

| **Characteristics** | **Practice** | | **P-value** | **Odds ratio (OR)**  **95% CI** |
| --- | --- | --- | --- | --- |
|  | **Good**  **n (%)** | **Poor**  **n (%)** |  |  |
| **Level of practice** | 136 (31.7) | 293 (68.3) |  |  |
| **Age** |  |  |  |  |
| Less than 22 | 74 (37.6) | 123 (62.4) | 0.01* | 1.6 (1.0-2.4) |
| 22 or above | 62 (26.7) | 170 (73.3) |  |  |
| **Sex** |  |  |  |  |
| Female | 72 (33.3) | 144 (66.7) | 0.46 | 1.1 (0.7-1.7) |
| Male | 64 (30) | 149 (70) |  |  |
| **Marital Status** |  |  |  |  |
| Unmarried | 126 (31.2) | 278 (68.8) | 0.35 | 1.4 (0.6-3.3) |
| Married | 10 (40) | 15 (60) |  |  |
| **Educational level** |  |  |  |  |
| Not completed two years | 65 (35.9) | 116 (64.1) | 0.11 | 1.3 (0.9-2.1) |
| Completed at least two years | 71 (28.6) | 177 (71.4) |  |  |
| **Monthly income of the family** |  |  |  |  |
| Below or equal to NRs. 40000 (≤USD 303.03) | 37 (27) | 100 (73) | 0.15 | 0.7 (0.4-1.1) |
| Above NRs. 40000 (>USD 303.03) | 99 (33.9) | 193 (66.1) |  |  |

*P-value statistically significant at the level of 0.05

**Correlation between knowledge, attitude and practice on dengue**

his study found that knowledge was positively correlated with an attitude (rka= 0.10). Similarly knowledge and practice were positively correlated (rkp=0.20) t. Meanwhileattitude was negatively correlated with practice (rap= -0.012). (Table 8)

*Table 8: Correlation between knowledge, attitude and practice on dengue*

|  | **Knowledge** | **Attitude** | **Practice** |
| --- | --- | --- | --- |
| Knowledge | 1 | 0.01 | 0.20 |
| Attitude | 0.01 | 1 | -0.12 |
| Practice | 0.20** | -0.12 | 1 |

** P-value statistically significantly associated at the level of 0.05

**Discussion**

Undergraduate students are a large group of the young population who can be a pivotal medium to disseminate the factual knowledge on dengue and play a vital role in raising awareness and promoting behavioral change in their communities (15).

This study showed that 84.8% of the students had good knowledge which is similar to previous studies conducted in Jamaica, Bangladesh and Malaysia (11,16,17). It can be due to the students being the major group particularly involved in the internet and social media to explore health issues. Another reason could be that students are also connected to different groups and sectors where they can access and exchange health related information from various sources. However, our study has different outcomes than reported with the similar studies conducted in Indonesia, India, Nepal, and Malaysia (13,18–20)**.** Thus, good knowledge among the students reflects that they have an adequate awareness level that can be used to mobilize communities. Our findings also demonstrate a knowledge and significantly wider practice gap for dengue among non-health undergraduate students, demonstrating an urgent need for improved public education and outreach about these issued in Nepal, which is experiencing a surge in cases and deaths due to dengue since 2022.

Most of the respondents were able to correctly identify typical symptoms of dengue. Among them, fever (90%) and headache (67.1%) were the most frequently mentioned symptoms and that is similar to the studies conducted in Jamaica, Bangladesh, Sri Lanka, India and Nepal (11,16,18,19,21).

Despite having good knowledge on dengue, more than half of the participants failed to mention other recognized symptoms of Dengue Fever (DF) such as pain behind the eyes and back pain. It could be because most participants were not experienced with the disease or had not witnessed a case from a close relative or a friend previously**.** One-third (34.5%) of respondents also believed that ordinary person-to-person contact can also transmit dengue, perhaps due to confusion with other communicable diseases such as COVID-19 and influenza.

o Our study reported there was a good knowledge l regarding dengue, however, few of them were unaware of the transmission of dengue. Only 38% could correctly identify the vector (Aedes mosquito) responsible for causing dengue. This findings was supported by the similar study conducted in Srilanka (21) and Nepal (15).

This study demonstrated a positive relation between knowledge with attitude, and practice. This finding is consistent with similar studies conducted in Nepal, Thailand and Jamaica (16,19,22) but contrary to studies conducted in Sri Lanka and Malaysia (17,21).

Most of the participants stated that television, social media and teachers were predominant sources on accessing information regarding dengue and that was similar to the findings reported by Jamaica, India and Malaysian (16,18,20). However our study revealed there was a negative correlation between attitude and practice and consistent with the study conducted in Brazil, Jamaica and Malaysia (16,17,23).

This study showed knowledge was significantly associated with age and educational level which is similar to the study done in Malaysia, Nepal and Indonesia (13,17,19). It might be due to the high health literacy level among the adults. This is supported by a study done in Malaysia that revealed that educational intervention was effective in creating positive awareness about dengue (20). . ge was associated with preventive practice regarding dengue . This implies that younger participants were more likely to engage in dengue preventive practices.

This study demonstrated that married groups had a better level of knowledge than their counterparts. It can be that married couples often share their daily experiences including health information, and if one partner has access to health-related information or works in the health sector, they may share their knowledge with family members.

This study highlighted that current KAP on dengue is crucial to designing effective community based interventions in order to improve prevention and control strategies regarding dengue. **Limitation**

Since this study was conducted web-based due the COVID-19 pandemic it was not possible to include participants who did not have access to the internet. The study relies on self-report measures, therefore it is possible that some participants may not have responded accurately. This study was limited with respect to environmental factors like climatic conditions, temperature, precipitation, and rainfall which can influence the prevalence of dengue.

**Conclusion**

Most students (84.8%) have good knowledge about dengue while only 25.9% and 31.7% of them have good attitude and practice regarding dengue. To control this young emerging disease, school health education can be the medium to help spread awareness on dengue in the community.

To bridge the gap between knowledge and practice on dengue and promote KAP on dengue, television and social media can be effective tools as these are major sources of information about dengue. As a discrepancy in knowledge and practice on dengue has been observed across the different age groups of students, tailored interventions for the different age groups can be devised to improve knowledge and encourage dengue prevention practices across the entire student population.

## Recommendation

The national government should support schools and universities in implementing health programs by providing guiding documents while also expanding its national dengue awareness campaign, targeting high-risk communities.

National government’s collaboration with local governments, Non-Government Organizations and concerned stakeholders can enhance dengue prevention and control, and support research and strategy development.

Local governments can help promote KAP on dengue by implementing targeted educational and awareness campaigns, tailoring interventions to specific age groups, supporting school-based preventive programs, and engaging in community-led actions for dengue prevention.. These measures can significantly reduce dengue morbidity and mortality, promoting public health and well-being.

Further studies on the epidemiology transitions of dengue, environmental factors and social factors need to be explored to better understand dengue prevention and management.

**Author Contribution**

SB: Conceptualization, Investigation, Methodology, Formal analysis, Writing-original draft preparation, Resources

MR: Formal analysis, methodology, writing-review and editing

RA: Methodology, writing: review and editing

NA: Manuscript review

KK: Writing-review and editing, Manuscript review

RP: Supervision, Manuscript review

**Funding Interest**

No funding was received for the study.

**Acknowledgment**

We would like to acknowledge all the faculty of the Central Department of Public Health, Institute of Medicine, Tribhuvan University, Nepal for their guidance during the research project. Our appreciation goes to individuals responding to the questionnaire for their communication and coordination.

**References**

1.	CDC. Centers for Disease Control and Prevention. 2022 (cited 2022 Dec 29). Dengue home | CDC. Available from: https://www.cdc.gov/dengue/index.html

2.	Dengue: Guidelines for Diagnosis, Treatment, Prevention and Control: New Edition. Geneva: World Health Organization; 2009.Avalaible from: PMID: 23762963.

3.	Bhatt S, Gething PW, Brady OJ, Messina JP, Farlow AW, Moyes CL, et al. The global distribution and burden of dengue. Nature. 2013 Apr;496(7446):504–7. Available from: doi: 10.1371/journal.pntd.0001760. Epub 2012 Aug 7.

4.	Guzman MG, Harris E. Dengue. The Lancet. 2015 Jan 31;385(9966):453–65.	Available form: https://doi.org/10.1016/S0140-6736(14)60572-9

5.	Pun SB. Dengue- An Emerging Disease in Nepal. J Nepal Med Assoc (Internet). 2011 Dec 31 (cited2022Dec31);51(184). Available from: https://www.jnma.com.np/jnma/index.php/jnma/article/view/33

6.	Dengue periodic outbreaks and epidemiological trends in Nepal | Annals of Clinical Microbiology and Antimicrobials | Full Text (Internet). (cited 2022 Dec 31). Available from: https://ann-clinmicrob.biomedcentral.com/articles/10.1186/s12941-018-0258-9

7.	Climate change threatens mountainous Nepal with a spike in dengue infections (Internet). (cited 2022 Dec 31). Available from: https://www.gavi.org/vaccineswork/climate-change-threatens-mountainous-nepal-spike-dengue-infections

8.	64b3dea63a1e4.pdf (Internet). (cited 2023 Aug 4). Available from: https://edcd.gov.np/uploads/resource/64b3dea63a1e4.pdf

9. Adhikari N, Subedi D. The alarming outbreaks of dengue in Nepal. Trop Med Health. 2020 Feb 7;48(1):5. Available from: DOI: [10.1186/s41182-020-0194-1](https://doi.org/10.1186/s41182-020-0194-1)

10.	national-guidelines-on-prevention-management-and-control-of-dengue-in-nepal.pdf (Internet). (cited 2020 May 6). Available from: [EDCD|National Guidelines of Prevention, Control & Management of Dengue in Nepal 2019](https://www.edcd.gov.np/resource-detail/national-guidelines-of-prevention-control-and-management-of-dengue-in-nepal-2019-updated).

11.Rahman MM, Khan SJ, Tanni KN, Roy T, Chisty MA, Islam MR, et al. Knowledge, Attitude, and Practices towards Dengue Fever among University Students of Dhaka City, Bangladesh. Int J Environ Res Public Health. 2022 Jan;19(7):4023. Available from: https://doi.org/10.3390/ijerph19074023

12.	Daniel WW, Cross CL. Biostatistics: A Foundation for Analysis in the Health Sciences. John Wiley & Sons; 2018. 720 p. Available from: ark:/13960/s27bt3sx3r3

13.	Harapan H, Rajamoorthy Y, Anwar S, Bustamam A, Radiansyah A, Angraini P, et al. Knowledge, attitude, and practice regarding dengue virus infection among inhabitants of Aceh, Indonesia: a cross-sectional study. BMC Infect Dis. 2018 Feb 27;18(1):96. Available from: https://doi.org/10.1186/s12879-018-3006-z

14.	Dhimal M, Aryal KK, Dhimal ML, Gautam I, Singh SP, Bhusal CL, et al. Knowledge, Attitude and Practice Regarding Dengue Fever among the Healthy Population of Highland and Lowland Communities in Central Nepal. PLOS ONE. 2014 Jul 9;9(7):e102028. Available from: <https://doi.org/10.1371/journal.pone.0102028>

15.	Jha R, Adhikari K, Shah DK, Ansari S, Dhungana G, Basnet S, et al. Knowledge and Awareness regarding Dengue among the Undergraduate health Science students of Dengue Hit region of Nepal. 2016 Feb 1;4:2320–7353. Available from: http://www.isca.in/

16.	Shuaib F, Todd D, Campbell-Stennett D, Ehiri J, Jolly PE. Knowledge, attitudes and practices regarding dengue infection in Westmoreland, Jamaica. West Indian Med J. 2010 Mar;59(2):139-46. PMID: 21132094; PMCID: PMC2996104.

17.	Hairi F, Ong CHS, Suhaimi A, Tsung TW, bin Anis Ahmad MA, Sundaraj C, et al. A Knowledge, Attitude and Practices (KAP) Study on Dengue among Selected Rural Communities in the Kuala Kangsar District. Asia Pac J Public Health. 2003 Jan 1;15(1):37–43. Available from: https://doi.org/10.1177/101053950301500107

18.	Jeelani S, Sabesan S, Subramanian S. Community knowledge, awareness and preventive practices regarding dengue fever in Puducherry – South India. Public Health. 2015 Jun 1;129(6):790–6. Available link: https://doi.org/10.1016/j.puhe.2015.02.026

19.	Phuyal P, Kramer IM, Kuch U, Magdeburg A, Groneberg DA, Lamichhane Dhimal M, et al. The knowledge, attitude and practice of community people on dengue fever in Central Nepal: a cross-sectional study. BMC Infect Dis. 2022 May 12;22(1):454. Available link: https://doi.org/10.1186/s12879-022-07404-4

20.	Wan Rosli, W.R., Abdul Rahman, S., Parhar, J.K. *et al.* Positive impact of educational intervention on knowledge, attitude, and practice towards dengue among university students in Malaysia. *J Public Health (Berl.)* **27**, 461–471 (2019). Available from: https://doi.org/10.1007/s10389-018-0971-z.

21.	Gunasekara TDCP, Velathanthiri VGNS, Weerasekara MM, Fernando SSN, Peelawattage M, Guruge D, et al. Knowledge, Attitudes and Practices Regarding Dengue Fever in a Suburban Community in Sri Lanka. 2015 Jul 14 (cited 2023 Mar 24); Available from: http://dr.lib.sjp.ac.lk/handle/123456789/1852

22.	Koenraadt CJ, Tuiten W, Sithiprasasna R, Kijchalao U, Jones JW, Scott TW. Dengue knowledge and practices and their impact on Aedes aegypti populations in Kamphaeng Phet, Thailand. Am J Trop Med Hyg. 2006 Apr;74(4):692-700. PMID: 16607007.

23.	Gonçalves Neto VS, Monteiro SG, Gonçalves AG, Rebêlo JMM. [Public knowledge and attitudes concerning dengue in the Municipality of São Luís, Maranhão, Brasil, 2004]. Cad Saude Publica. 2006 Oct;22(10):2191–200. Available from: 10.1590/s0102-311x2006001000025.
