# Supplementary material for: Knowledge, attitude, and practice regarding dengue among non-health undergraduate students of Nepal
Source: PLoS Negl Trop Dis. 2024 May 28;18(5):e0012222. doi: 10.1371/journal.pntd.0012222 (PMC11161042; doi:10.1371/journal.pntd.0012222)
Supplement: Attachment — Submitted filename: Reviewer Response (R2) -editor response.docx [file pntd.0012222.s006.docx]

**Title: Knowledge, attitude, and practice regarding dengue among non-health undergraduate students of Nepal.**

To

The Editorial Board,

PLOS-Neglected Tropical Diseases

Thank you for the review and for providing feedback/suggestions.

We have addressed the comments and made necessary changes/corrections in the updated manuscript entitled **“Knowledge, attitude, and practice regarding Dengue among non-health undergraduate students of Nepal.” [Ms. Ref. No: PNTD-D-23-01001].** Both tracking and a clean version of an updated version of the manuscript are also provided along with this response.

Thank you.

Sincerely,

Manish Rajbanshi

Corresponding author

**Reviewer response**

**You need to better describe how you made contact with students and incorporate your response to Reviewer #2 about the response rate to the questionnaire.**

*The contact with the students was made through social media (i.e. Facebook, Gmail, WhatsApp, and Viber). This information has been updated in the “Data Collection” section of the manuscript.*

***Editor Response:*** *Although the added text is helpful, there are still a few details missing. What would be very useful if you included the recruitment message in your supplemental information.*

*The critical questions are: 1) How did you select the people that your contacted, I remember you indicated 550 (previous responses)? Did you send the google form link to all registered students over 18 years of age in all 11 provinces. How did you get their gmail, facebook, what App or viper contact information? Are these students who were facebook friends, or subscribed to university news letters or part of University What’s app groups? How you did this is very important to understand.*

**•	Report the range of age, from your response one still asks if the range is from 19-22 or 19-25. For example, in Table 1 instead of Less than 22 and 22 or above include the ranges, i.e. 19-21 versus 22-25.**

*We apologize for the inconvenience. We placed age in range in Table no. 1 i.e., 18-21 and 22-37 and as necessary.*

***Editor response:*** *THANKS, this provided some additional insight about the older age group, not elderly but persons in the 30-37 age range may have very different experiences than younger age groups.*

**•	For provinces, perhaps you can included Bagmati and Lumbini (assuming they are closest to the named universities) and Other (you can list the as a footnote).**

*Thank you for providing an opportunity to share with you. This study included undergraduate students from four national universities in Nepal. As well, this study was flexible to include undergraduates across all the provinces of Nepal and was not limited to particular provinces.*

***Editor response:*** *See some of my edits to the word version of your article. Okay as is in the text but the disproportionate number of participants from Bagmati and Lumbini Provinces deserves some explanation in the discussion of the manuscript. This difference does not seem to be associated with population size, but Reviewer #1 made an important point about understanding how representative your sample is of these non-health students across Nepal.*

**•	When updating this sections, please give the reasons why you selected these 4 universities and what segment of the Nepalese population they are likely to represent.**

*Thank you for sharing with us. Among 11 national universities of Nepal, these four universities consisted of more than 80% of the undergraduate and postgraduate students of Nepal i.e., Tribhuvan University (75.94%), Purbanchal University (6.23%), Pokhara University (6.91%) and Kathmandu University (4.15%). This information is also updated in the revised manuscript.*

*Reference:* [*https://edusanjal.com/blog/nepal-education-in-numbers-2019/*](https://edusanjal.com/blog/nepal-education-in-numbers-2019/)

***Editor response:*** *So I had it wrong, again check your text to make sure that my edits now reflect what when on. The document in the link is very helpful, you might cite it in your article somewhere. A couple of questions for clarification: 1) Makes sense that most of your respondents are from the Top 4 universities, this appears to track enrolment numbers, but where are these Universities located? 2) Does the Province question relate to where the student is from originally or the location of their university?*

**•	State in the methods that the form was administered in English.**

*Thanks for sharing with us. This study used a self-administered tool in the English language and it has been updated in the revised manuscript.*

***Editor response: Thanks!***

**•	Add your response to Major comment from Reviewer #1. That is add a section to the methods on validation and pretesting of the study instrument.**

*Pleased to share this with you. The pretesting was done among around 10% of the sample size of the study (n=45) via online form. Cronbach’s Alpha was used to assess the reliability coefficient which is a measure of the internal consistency of the questionnaire. The result showed that Cronbach’s Alpha coefficients of KAP domains were 0.80, 0.72, and 0.74 respectively.*

*This information has also been added in the ‘tools and measures’ section of the revised manuscript.*

***Editor response: Thanks!***

**•	Include the definition of non-health students (also in the response to Major comments Reviewer #1) in this section.**

*Thank you for providing an opportunity to share with you. Non-health students refer to “Students other than health science such as from engineering, agriculture, management, forestry, veterinary, arts, and information technology backgrounds were considered as non-health students in this study.*

***Editor response: Thanks!***

**•	Ethical clearance: I am not as concerned about this, but all individuals who worked with identifiable information (PII) should have approval from each person’s institution or have those institutions defer to Tribhuvan University. Usually this is done through what is called a relying agreement or a letter indicating this was okay with each of the other institutions.**

*Thanks for the opportunity to improve our manuscript. The study was based on self-reported online data collection and e-consent was obtained from each participant on the first page of the Google Form. In addition, code was used for each study participant to maintain the confidentiality of their information. Therefore, approval or letter of agreement from additional universities was deemed unnecessary.*

***Editor response:*** *You have not really addressed this question, but if accepted the Journal office will ask you to provide more details on this issue.*

**•	In the Data management and analysis section, you should state that you carried out a multivariate analysis, but no significant differences were found and will not be discussed more.**

*Thank you for your suggestion. A multivariate analysis is also mentioned in the ‘Data and analysis’ section stating no significant difference between KAP and individual characteristics. Also, confounding characteristics to the outcome were mentioned in the limitation of this study.*

***Editor response:*** *I continue to have some serious concerns about your statistical analysis. You did Chi-square tests which are to test of independence they do not test for “associations”. That is a significant chi-square test says there is a difference but suggests no causation or association, you can simply note that the proportions are not the same between the groups. If you conduct Univariate logistic regression for each variable of interest and only include those that are significant or traditionally near significant (p<0.1 or some select <0.2), you then build a multi-variate model by backward or forward selection. I suggested you get some statistical help on the last review and believe you need to do so for this article to be published. Chi-square analysis is not sufficient. A proper statistical model will provide important insights and help you interpret your findings.*

**•	In addition, to the inclusion of the questionnaire, you should indicate what you scored as a “correct” answer. In regard to this in Table 2, perhaps you could highlight statements that are incorrect in another color to distinguish with statements that are true. I will put the incorrect statements in red.**

*Thank you for the suggestion, we have accordingly highlighted the correct response (in green) in table no. 2 in the tracking version manuscript.*

***Editor response: Thanks.***

**Other comments and observations**

**•	“youngest” emerging infection. Note sure what you mean by that. Do you a “recent”?**

*Thank you for your suggestion. The ‘youngest emerging infection; is changed to ‘recent emerging disease’ in the revised manuscript. Now, it can be read as “Dengue poses a significant public health concern worldwide. It is identified as a recent emerging infectious disease in Nepal.” in the revised manuscript.*

***Editor response: Thanks.*** *You need to be sure you make the same change throughout the manuscript.*

**•	Abstract – see some of my edits in yellow highlight or by comment. I have some concerns about Lines 40-42 and Lines 43-49.**

*Thank you for your suggestion in the corresponding lines. We would like to incorporate the changes in the edits***.**

***Editor response:*** *The additional information you provided helps, but to put each of the variables you are testing on the same scale I suggest you run your correlation analysis on K, A, P – scores as a percentage rather than the absolute score. Not sure it will change the outcome, but I think this would be more appropriate. Also you should think about the magnitude of the correlations, for example -0.01 is so close to zero even if it is significant, it seems meaningless.*

**•	Introduction: see edits in yellow highlight.**

*Thank you for your suggestion in the lines. We would like to incorporate the changes in the edits.*

***Editor response: Thanks.***

**•	Methods: While you clearly describe how knowledge was scored (80%), it is not clear how you scored Attitudes or Practices. Because of the Likert scale it would be different for Attitudes. You do not describe how “Not sure’s” are handled. You should clearly state for each section the maximum number of points awarded.**

*Thank you for the review. We have now added the information about how we handled the scoring on KAP as* ***“****Evaluation of knowledge, attitude, and practices were done by assigning ‘1’ for correct /positive response and ‘0’ for incorrect/negative response. Participants were categorized into having good knowledge, good attitude, and good practice if they had scored 80% or higher (score ≥ 19) or above, and poor knowledge if they had scored below 80% (score <19) in this study.*

*Participants were asked to rate their attitude on dengue using a five-point Likert scale. The scale ranged from “Strongly disagree” to “Strongly agree”. For each question, participants were assigned scores of 1, 2, 3, 4, and 5 corresponding to the following responses: Strongly Disagree, Disagree, Not Sure, Agree, Strongly Agree. Based on the total score, participants were categorized as poor (score <24) and good attitudes (score ≥ 24).*

*There was a total of 19 questions related to practice. Each participant was assigned a score of ‘1’ for positive practice and ‘0’ for negative practice. Based on the total score, participants were categorized as poor (score <16) and good practice (score ≥ 16)”.*

***Editor response: Thanks this was important to include.*** *My only suggestion is that you might consider using 15 instead of 16 as your cut off as 78% is closer to 80% than 84% making it more comparable to both the K and A scores. I also was comforted to see that you’re a-score reflects people who said both strongly agree and agree.*

**•	Methods: We need a bit of information, based on the previous question on the correlation analysis. Were all percentages or was the correlation analysis done with absolute scores. I would like to see a regression of these data. It is difficult to believe the negative correlation of attitudes with practices, we need to understand that. There is often a gap between knowledge and practice, but I think you might consider a knowledge-Attitude combined score.**

*Thank you for your review. The correlation analysis was done using absolute scores. While studies showing negative correlation between attitude and practice on dengue is not very common, studies such as the one in* [*IJERPH | Free Full-Text | Perceived Risk for Dengue Infection Mediates the Relationship between Attitude and Practice for Dengue Prevention: A Study in Seremban, Malaysia (mdpi.com)*](https://www.mdpi.com/1660-4601/19/20/13252) *-* [*https://www.mdpi.com/1660-4601/19/20/13252*](https://www.mdpi.com/1660-4601/19/20/13252) *has demonstrated the mediating effect of perceived dengue risk in the relationship between attitude and dengue prevention practices. This shows that practice on dengue is not directly influenced by attitude on it.*

*[NOTE: The output of the correlation analysis is also provided as a supplementary file.]*

***Editor response:*** *I suggest that you use a similar analysis approach as the article you mention above, no Pearson correlation analysis, nor did I see a negative correlation. Clearly there is a big gap between both Knowledge, Attitude, resulting in appropriate Practices, but a negative association between attitude and practice implies that people who agree with actions are less likely to practice them than those who don’t have appropriate attitudes toward action. Your results suggestion people attitudes about dengue don’t make much difference in their practices. You need to improve your statistical approach.*

**•	Methods: Need to mention you tried a multi-variate model. My strong suggestion would be to ask for some statistical help on this. Additionally, you should look into logistic regression for your univariate analysis as well. Chi squares to test for independence of two variables but not an association.**

*Thank you for your suggestion. A multivariate analysis is also mentioned in the ‘Data and analysis’ section stating no significant difference was observed between KAP and individual characteristics.*

***Editor response:*** *See my response above, but Chi square to not measure associations, you need to first do univariate logistic regression, one variable at time, then try and build a multivariate model based on those results. You provide no information on how you built or analysed your multivariate model. I think you need to try your analysis again.*

**•	Results: Is a bed net viewed as a preventative measure for dengue? Aedes aegypti is a day biter so bed nets really are not protective.**

*Thank you for your review, while it is true that Aedes aegypti bites during the day, bed nets can still offer some protection even for day nappers.*

***Editor response:*** *I will accept that, but would not make this a key component of an anti-dengue campaign.*

**•	Results: Age and education level look confounded here. You could try a stratified analysis looking to find if you where you look at young people with more education, older people with less education, young people with less education and older people with more education.**

*Thank you for your observation! It appears age and education level might be confounded in our current analysis. While a stratified analysis could be helpful, delving into this level of detail might be beyond the scope of our present research. We have mentioned this in the limitation section.*

***Editor response:*** *As a reviewer and editor, I challenge you to do this. In fact, I might treat age as a continuous variable (regression) and at the very least do a graph of age on the X-axis and your K, A, and P scores on the y-axis. You might see some interesting patterns (maybe not). You have the data this should not be that time consuming, and it will help you interpret your data. This is an analysis you can do with the data you have, I would have done it myself, if I could have accessed your raw data files which are in SPSS format. Please save that file as csv or txt so anybody could look and check your analysis. This is now a requirement from PLOS NTD.*

**•	Results: You can’t do anything about it now, but I don’t like your questions associated with breeding sites (larval habitats). No questions about mosquito larvae and if people get they become adult mosquitoes. Also don’t like reference to “broken” containers. Aedes is very happy in intact containers.**

*Thank you for your review and comments.*

*OK*

**•	Results: LINES 195-199. WHAT IS A POOR ATTITUDE ON DENGUE AND HOW WAS THAT CALCULATED? IF you add strongly agree with agree, seems like most respondents have an appropriate attitude about dengue, although some of these questions are more about knowledge.**

*Thank you for the review. We have now added the information about how we handled the scoring on KAP in the methodology section.*

***Editor response: Much clearer now.***

**CRITICAL ISSUES.**

**•	How were attitude and practice scores calculated.**

Thank you for the review. We have now added the information about how we handled the scoring on KAP in the methodology section.

**Resolved.**

**•	Correlation analysis seems wrong – I can not access the data set which should be saved in csv or txt format.**

Thank you for the review. The output of the correlation analysis is also provided as a supplementary file.

My question relates to what numbers you used to produce the output and again please save data files in csv or txt format, excel would also be okay, but I don’t use SPSS.

FINAL COMMENT:

YOU NEED TO ADDRESS THE STATISTICAL CONCERNS I BROUGHT UP. YOU SHOULD BE ABLE TO EXAMINE THE RELATIONSHIP OF AGE MORE CLOSELY AND YOU NEED SOME KIND OF REGRESSION (LOGISTIC) OR A NON-PARAMETRIC EQUIVALENT. CHI-SQ is not sufficient.
